# Effect of* Terminalia catappa *Linn. on Biofilms of* Candida albicans* and* Candida glabrata* and on Changes in Color and Roughness of Acrylic Resin

**DOI:** 10.1155/2019/7481341

**Published:** 2019-07-07

**Authors:** Letícia Machado Gonçalves, Petrus Levid Barros Madeira, Rafael Soares Diniz, Rammon Farias Nonato, Fabiana Suelen Figuerêdo de Siqueira, Eduardo Martins de Sousa, David Cardoso Sandes Farias, Flaviane Maria Galvão Rocha, Carlos Henrique Lopes Rocha, Andréa Dias Neves Lago, Cristina de Andrade Monteiro

**Affiliations:** ^1^Department of Dentistry, Post-Graduate Program in Dentistry, CEUMA University, Sao Luis, Maranhao, Brazil; ^2^Department of Dentistry, CEUMA University, Sao Luis, Maranhao, Brazil; ^3^Laboratory of Applied Microbiology, Post-Graduate Program in Microbial Biology, CEUMA University, Sao Luis, Maranhao, Brazil; ^4^Graduate Program in Dentistry, Federal University of Maranhao. UFMA, Sao Luis, Maranhao, Brazil

## Abstract

This study aimed to investigate the effect of the n-butanol fraction of* Terminalia catappa *Linn., (FBuTC) on biofilm of* Candida albicans* and* Candida glabrata*, as well as changes in color and roughness of polymethyl methacrylate resin (PMMA). The susceptibility of* C. albicans* and* C. glabrata* to FBuTC was evaluated by means of the Minimum Inhibitory and Minimum Fungicidal Concentration (MIC and MFC). PMMA acrylic resin discs (N= 108) were fabricated. For the susceptibility tests, biofilms of* C. albicans* and* C. glabrata* were developed on discs for 48 h and immersed in phosphate-saline buffer solution (PBS), 1% sodium hypochlorite (SH 1%), or FBuTC at MIC, 5xMIC, or 10xMIC. For the color and roughness change tests, the discs were immersed in distilled water, SH 1%, or FBuTC in the concentrations of 0.25 mg/mL, 2.5 mg/mL, or 25.0 mg/mL. After 28 days of incubation, color change was evaluated by spectrophotometry and roughness, by using a profilometer. The biofilms were investigated by one-way ANOVA and, the color and roughness changes (two-way ANOVA and the Tukey test; *α*=0.05). For both MIC and MFC the value of 0.25 mg/mL of FBuTC was observed for the planktonic cells of* C. albicans* and* C. glabrata*. Exposure to FBuTC at 10xMIC had a significant effect on the biofilm of* C. albicans*, showing a reduction in cell counts when compared with PBS, (*p*=0.001). For the biofilm of* C. glabrata*, the MIC was sufficient for significantly reducing the cell count (*p*<0.001). No important changes in color and roughness of the acrylic resin were observed, even after 28 days, irrespective of the concentration of FBuTC used (*p* >0.05). It could be concluded that the immersion of acrylic resin for dental prosthesis in FBuTC was effective in reducing the biofilms of* C. albicans* and* C. glabrata* without evidence of change in roughness and color of this substrate.

## 1. Introduction

For decades the public oral health policy in underdeveloped or developing countries was based on tooth extraction for the prevention of pain, caries, and infection, among other oral problems. This curative practice is reflected in the contemporary population, in which we have found a high number of partially or completely edentulous individuals [1]. This population is generally rehabilitated with removable dental prostheses, which are fabricated of poly(methyl methacrylate)-based acrylic resin, due to its good esthetic qualities and excellent cost-benefit ratio [2].

However, acrylic resin is easily colonized by oral endogenous bacteria and* Candida *spp. and, eventually, by extraoral species, such as* Staphylococcus* spp. or members of the Enterobacteriaceae family. This microbial reservoir may be responsible for denture-related stomatitis and aspiration pneumonia, a life-threatening infection, especially in geriatric patients [3]. In view of this context, adequate control of the biofilm formed on denture surfaces is of outstanding importance, not only for oral health, but also for the general health of denture-wearers.

The method most used for biofilm control is brushing with toothpaste, because it is simple and low cost [4, 5]. However, effective removal of biofilm by brushing is questionable, because dental prosthesis surfaces are irregular and porous, and although brushing is the most used method, patients with motor coordination problems have difficulty with performing this procedure adequately [6]. Moreover, the continual brushing of acrylic resin could lead to its abrasion by friction of the toothbrush bristles, which could increase the surface roughness of the denture, and this would work by serving as a niche for microorganisms [7].

By virtue of these limitations, the use of disinfectant or chemical cleaning solutions has been proposed as an important additional method for eliminating the microorganisms, because they produce more efficient results, especially in geriatric patients [8]. Sodium hypochlorite, for example, is an extensively used disinfectant solution that promotes efficient cleaning; however, it has the disadvantages of unpleasant taste and smell, changing the strength and color of acrylic resin over the course of time [9], while chemical cleaning solutions have an effervescent effect when dissolved in water, resulting in an alkaline solution of hydrogen peroxide [10]. Some studies have investigated the effect of cleaners on biofilms formed on denture surfaces [11]; however, they have observed that this method still fails to remove biofilms of* Candida* spp. [11, 12].

In situation in which denture stomatitis has become a persistent infection, studies have recommended the use of antifungal agents, such as the polyenes and azoles [13]. However, the indiscriminate use of these agents may contribute to the selection of resistant strains of* Candida* spp., especially of virulent species such as* C*. glabrata [14, 15].

Considering the limitations associated with chemical-mechanical control of biofilms, and the selection of resistant strains, the quest for antifungal substances coming from natural inputs has received renewed attention. Nevertheless, the search for active principles for formulation of efficient products with low toxicity [16, 17], which are inexpensive and provide the population with new options, consists of a challenging task.

In this context, medicinal plants have shown to be promising for the prevention and treatment of fungal infections [16]. Among these,* Terminalia catappa *Linn., also known as Indian or tropical almond tree, has attracted the attention of researchers because it has exhibited various biological activities and properties such as antioxidant, [18] antiviral [19], anti-inflammatory [20], and antimicrobial action [21]. Previous studies have suggested that some fractions (i.e., n-butanol) obtained from the leaves of the Indian (tropical) almond tree are especially effective against bacteria [22] and fungi [23], but little is known about the effects of this fraction against biofilms of* Candida* spp. Furthermore, the main disadvantages of the prosthesis cleaning methods are considered to be discoloring of the acrylic denture base and possible changes in surface roughness [7, 24].

Diverse natural products obtained from plants used in popular medicine have been used as basic structures for synthesis and development of new medications. The value of these natural products to society and the economy is incalculable, which encourages researches that seek active principles for the prevention and treatment of infections [16–18, 25–29]. The aim of this study was to investigate the effect of the n-butanol fraction of* Terminalia catappa *Linn. (FBuTC) on biofilm of* Candida albicans* and* Candida glabrata*. In addition, changes in color and roughness of polymethyl methacrylate resin (PMMA) were investigated, after immersion in the n-butanol fraction of* Terminalia catappa *Linn. (FBuTC).

## 2. Materials and Methods

### 2.1. Experimental Design

The susceptibility of planktonic cells of* C. albicans* (ATCC 90028) or* C. glabrata* (ATCC 2001) to FBuTC was evaluated by means of Minimum Inhibitory Concentration (MIC) and Minimum Fungicidal Concentration (MFC) tests considering the effects on the acrylic resin substrate, these were measured by means of surface roughness and color change tests. For the tests, poly(methyl methacrylate)-based acrylic resin (PMMA) discs were fabricated in accordance with the manufacturer's instructions, and their roughness was standardized. For the susceptibility tests, biofilms of* C. albicans* or* C. glabrata* were developed on the discs for 48 hours. The biofilms were immersed in the following solutions for 10 minutes: phosphate-saline buffer (PBS, negative control), 1% sodium hypochlorite (1% SH, positive control), or FBuTC in the concentrations of MIC, 5xMIC, or 10xMIC. The biofilms were investigated in regard to cell counts and all the experiments were conducted in triplicate, in three different time intervals. Considering the effects on the acrylic resin substrate, the discs were immersed in the treatments and surface roughness and color change tests were performed 28 days after immersion in the different concentrations of FBuTC. Exposure to distilled water and 1%Sh were used as controls. All the experiments were performed in triplicate in the different time intervals.

### 2.2. Collection, Botanical Identification and Preparation of FBuTC

The* Terminalia catappa *Linn. leaves were cultivated in an experimental field of the Federal University of Maranhão, São Luiz, Maranhão, Brazil. The sample was collected from September 2018 to November 2018. The exsiccatae were prepared and sent to the “Herbário Ático Seabra” of the Federal University of Maranhão for botanical identification. The leaves were dried separately in an oven with air circulation at 37°C for 48 hours, followed by being ground by means of a mill. The dry, ground material (approximately 200 g) was macerated with approximately 800 mL of 70% ethanol at ambient temperature for 24 hours. This process was repeated four times, and the extract obtained was filtered and then concentrated using a rota-evaporator. The dry residue was suspended in MeOH/H_2_O (80:20, v/v), and the samples were sequentially submitted to liquid-liquid fractioning with hexane (Merck, Darmstadt, Germany), followed by ethyl acetate (Merck, Darmstadt, Germany) and n-butanol (Merck, Darmstadt, Germany), resulting in three fractions: hexane (FHexTC), ethyl acetate (FAcOEtTC), and n-butanol (FBuTC) fractions. The FBuTC was concentrated using the rota-evaporator and stored in an amber flask until the experiments were performed.

### 2.3. Susceptibility Tests

To evaluate the susceptibility of the planktonic cells to FBuTC, MIC, and MFC analyses were used. As control group, a fluconazol solution was prepared, the action of which has been well established in the literature. For reactivation of the microorganism and preparation of the inoculum, two reference strains* C. albicans* (ATCC 90028) and* C. glabrata* (ATCC 2001) were used. Each strain was reactivated in its original culture in Sabouraud dextrose agar (SDA) at 37°C, for 24 hours. To prepare the inoculum, colonies were suspended in Yeast Nitrogen Base (YNB) broth enriched with 50 mM of glucose. This set was incubated at 37°C for 20 hours, and after this was centrifuged (5000 rpm, 4°C) and washed with PBS. An aliquot of centrifuged cells was transferred to a tube containing saline solution and the turbidity of this content was adjusted with the use of a spectrophotometer (Spectronic 20; Bausch & Lomb, Rochester, NY, US) to assure a suspension of ≈10^7^ cells/mL (optical density = 0.25 at 520 nm).

The MIC was determined by the microdilution method in broth recommended by the Clinical and Laboratory Standards Institute (CLSI) [30]. From the initial concentration of FBuTC, which was defined in pilot tests, serial dilutions were made in 96-well plates. The wells containing the different solutions of FBuTC, controls (positive and negative) and inoculum were incubated at 37°C for 48 hours. The test readout was made by visual comparison, and the MIC corresponded to the lowest concentration that prevented visible growth of the planktonic cells. Each concentration of the previous test that presented no visible growth was inoculated on an SDA plate. After 24 hours of incubation at 37°C, the MIC readouts were made, based on the growth of the controls, and the MFC was considered the lowest concentration of the extract that prevented fungal growth (≥ 99.9%.).

### 2.4. Acrylic Resin Specimen Fabrication

A total of 108 circular discs (10 mm in diameter x 2 mm thick) were made of pink PMMA-based resin (QC 20, Dentsply Ind e Com. Ltda.), using a stainless steel muffle containing orifices in these dimensions, in accordance with the manufacturer's instructions (pressure of 1 T, 20 minutes at 100°C). Afterwards, the discs were finished with water abrasive papers #° 320, 400, and 600 in a horizontal polishing machine (Arotec; São Carlos, SP, Brazil). Subsequently, they were stored in distilled water at 37°C for 48 hours to allow elimination of residual monomers. To standardize the surface roughness, this was measured with a profilometer (Surfcorder SE 1700; Mitutoyo) with an active tip diameter of 2 *μ*m, precision of 0.01 *μ*m and speed of 0.5 mm/s under a pressure of 0.07 N. Three measurements were made, and the arithmetic mean was calculated, thus defining the roughness value. For standardization purposes, a variation of more or less 5% of the mean value was used.

### 2.5. Development and Analysis of Biofilms

For biofilm formation, acrylic resin discs (n = 54) were allocated to 24-well plates and exposed to the previously adjusted inocula of* C. albicans* or* C. glabrata* (i.e., a suspension of ≈10^7^ cells/mL). The plates were incubated under constant agitation at 37°C, for 90 min, the time corresponding to the cell adhesion phase. Afterwards the discs were transferred to wells containing YNB medium supplemented with 100 mM and maintained at 37°C for 24 hours to allow biofilm development. This process was repeated until the biofilm completed 48 hours. After completing 48 hours, the biofilms were immersed in the following solutions for 10 minutes: phosphate-saline buffer (PBS, negative control), 1% sodium hypochlorite (1% SH, positive control), or FBuTC in the concentrations of MIC, 5xMIC, or 10xMIC. Cell counts were made by means of serial dilutions. For this purpose, the biofilm developed on the discs was sonicated (7 Watts, 30 s) in saline solution for cell disaggregation. The suspension obtained was submitted to decimal serial dilution, and the product of each dilution was inoculated on SDA plates in triplicate. The plates were incubated at 37°C for 24 hours. The colonies were quantified visually, and the result was expressed in cells/mL.

### 2.6. Effect on Acrylic Resin

The acrylic resin discs (n = 45) were immersed in distilled water (negative control), 1% Sodium Hypochlorite (1%SH, positive control), or FBuTC in the concentrations of 0.25 mg/mL and 2,5 mg/mL to 25.0 mg/mL. The discs were incubated at 37°C for 28 days. The immersion medium was changed daily. Afterwards, the discs were washed in distilled water and dried with absorbent paper. The tests were performed after time intervals of 7, 21, and 28 days of immersion. All the tests were performed in triplicate, in three different time intervals (n=9).

For perception of color change, the discs were placed in a silicone mold with an orifice, for the purpose of adapting a portable spectrophotometer to the mold (EasyShade Advance 4.0; Wilcos, Germany). This mold was used for the purpose of allowing precise repositioning and measuring of the color of the disc surface. The color measurements were obtained by using the CIEL*∗*a*∗*b*∗* system. The total color change (ΔE) was calculated using the following formula: ΔE*∗*= (ΔL*∗*)^2^ + (Δa*∗*)^2^ + (Δb*∗*)^2^. Values of ΔE>3.7 were considered clinically imperceptible [28].

For the surface roughness analysis, three readouts were made on each disc by using a profilometer (Surfcorder SE 1700; Mitutoyo). The roughness of each disc was calculated to obtain the arithmetic mean value. The change in surface roughness (ΔRa) was obtained by the difference in roughness after immersion and the initial roughness values (baseline).

### 2.7. Statistical Analysis

The susceptibility test results were statistically analyzed by the SAS/LAB Software (SAS Software, version 9.0; SAS Institute Inc., Cary, NC, USA). Normal distribution of the data was previously adjusted, and when necessary, the data were transformed as suggested by the software. The cell count data were analyzed by the one-way ANOVA, followed by the Tukey test. The color and surface roughness change data were analyzed by two-way ANOVA for repeated measures, followed by the Tukey test, in which the periods of treatment and type of immersion were considered study factors. The level of significance was established at 5% for all the tests.

## 3. Results

### 3.1. Susceptibility Tests

Both the MIC and MFC values of 250.0 *μ*g/mL of FBuTC were observed for the planktonic cells of* C. albicans* and* C. glabrata*, demonstrating a fungicidal pattern of behavior. The values obtained for the control group with fluconazol were 0.5 *μ*g/mL and > 64 *μ*g/mL for MIC and MFC, respectively.

### 3.2. Analysis of Biofilms

**Figure 1:**
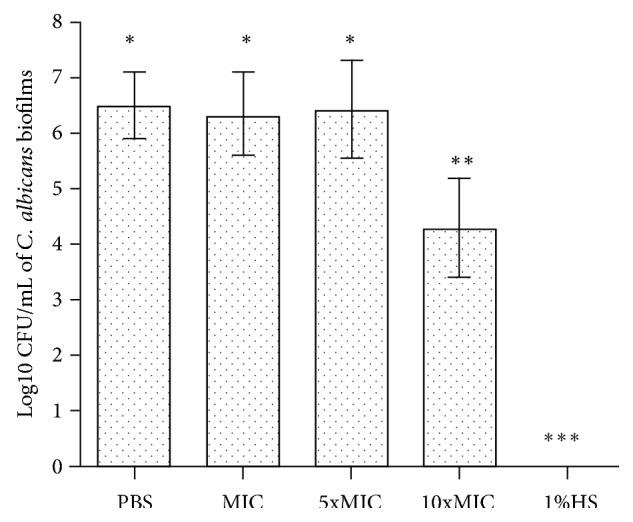
Effect of FBuTC on cell counts of biofilm of* C. albicans*. The presence of different symbols (*∗*, *∗∗* and *∗∗*) indicated statistically significant difference between the groups (one-way ANOVA followed by the Tukey test,* p* < 0.05).

For the biofilm of* C. albicans*, exposure to FBuTC for 10 minutes at 10xMIC had significant effect, reducing the viable cell count when compared with the negative control (*p* = 0.001; Figure 1). The MIC and 5xMIC were insufficient for reducing the cells of biofilms of* C. albicans* (*p* > 0.05).

**Figure 2:**
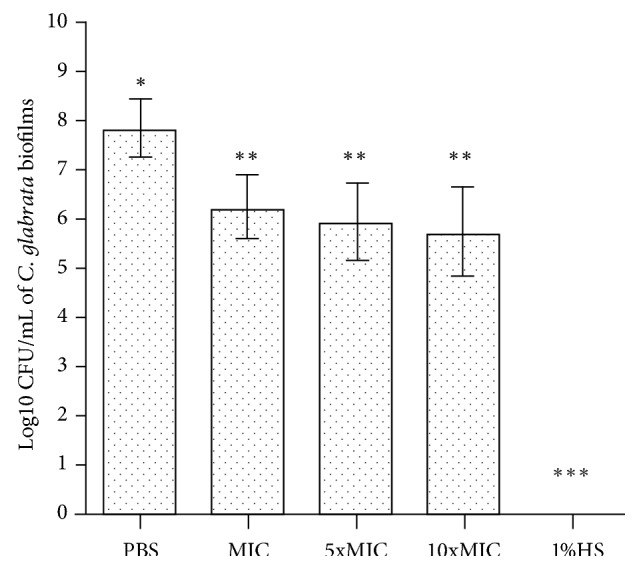
Effect of FBuTC on cell counts of biofilm of* C. glabrata*. The presence of different symbols (*∗*, *∗∗* and *∗*) indicated statistically significant difference between the groups (one-way ANOVA followed by the Tukey test,* p* < 0.05).

Whereas, for the biofilm of* C. glabrata*, the MIC was sufficient for significantly reducing the cell count when compared with the negative control (*p*<0.001; Figure 2), for both biofilms, the positive control group (1%SH) presented fungicidal activity, completely eradicating the fungal cells.

### 3.3. Effect on Acrylic Resin

**Figure 3:**
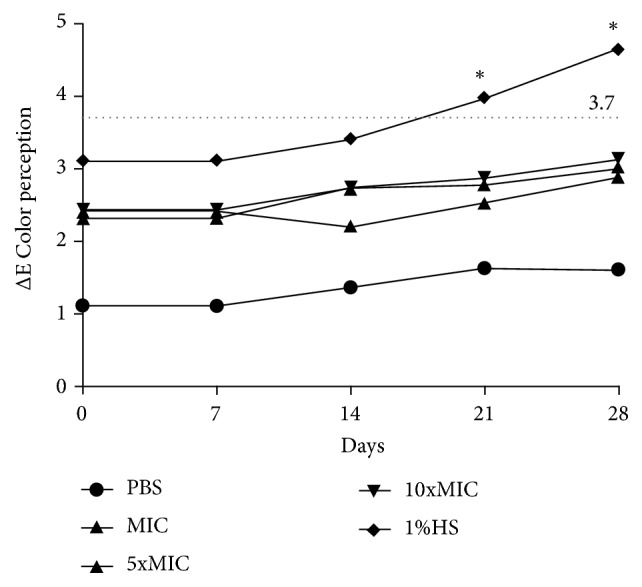
Color change values (ΔE) after 28 days of immersion of acrylic resin specimens on FBuTC. The presence of symbol (*∗*) indicated statistically significant difference between the groups (two-way ANOVA for repeated measures followed by the Tukey test,* p* < 0.05).

Considering the color change values (ΔE), when compared with the distilled water group, statistical differences were observed for both the immersions in 1%SH and FBuTC, in all the time intervals of immersion (*p* < 0.05). In spite of this variation, all the ΔE values obtained of the immersions in FBuTC were < 3.7; thus they were classified as being imperceptible. On the other hand, clinically perceptible changes were detected in the group with 1%SH as from the 21st day of immersion (*p* < 0.05, Figure 3). Moreover, pilot studies demonstrated that solvent (n-butanol) had no effects on acrylic surfaces (data not shown).

**Figure 4:**
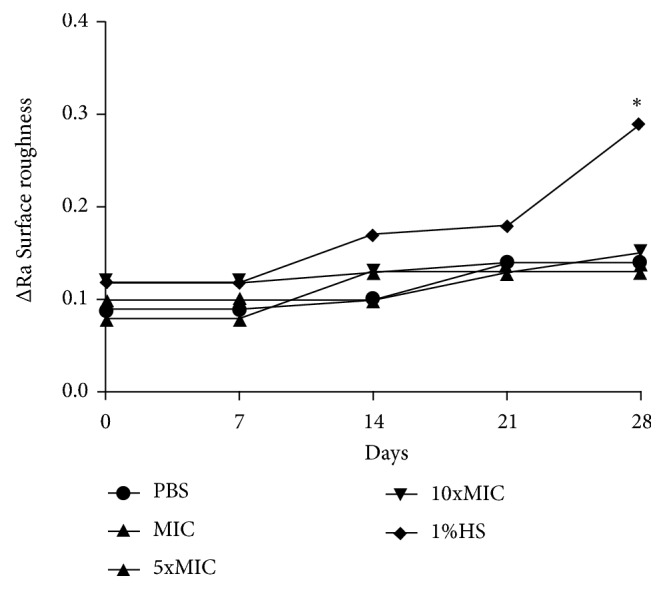
Surface roughness values (ΔRa) after 28 days of immersion of acrylic resin specimens in FBuTC. The presence of symbol (*∗*) indicated statistically significant difference between the groups (two-way ANOVA for repeated measures followed by the Tukey test,* p* < 0.05).

As regards the surface roughness values, there was no statistically significant difference between the group with distilled water and FBuTC in any of the concentrations (*p* < 0.05, Figure 4). Significant changes in roughness were detected only for Group 1%SH after 28 days of immersion

## 4. Discussion

In this study, the effect of the n-butanol fraction (FBuTC) of* Terminalia catappa *Linn. on biofilms of* C. albicans* or* C. glabrata* developed on acrylic resin was investigated. This* in vitro* condition simulated the biofilm developed on the surface of a removable dental prosthesis, in an endeavor to evaluate the potential of FBuTC as an auxiliary method for the control of denture stomatitis.

As a starting point for the test against biofilms, the susceptibility of planktonic cells of* C. albicans* and* C. glabrata* to FBuTC was investigated. By means of this test, coincident MIC and MFC values were observed (i.e., 0.25 mg/mL of 250 *μ*g/mL), showing evidence of the fungicidal potential of the extract against both species, which corroborated the findings shown in the literature [23]. We emphasize that the MIC values were obtained in tests that evaluated the cells in their planktonic form. However, in the oral cavity, these cells are organized in biofilms on the surface of dental prostheses, which makes them relatively more resistant to the actions of antifungal agents [31–33].

In this study, the antifungal effect of FBuTC was evaluated in mature biofilms, (i.e., 48 hours of development), and the immersion time of 10 minutes was chosen considering the time recommended for the positive control, 1%SH [34]. Moreover, the concentrations of 5xMIC and 10xMIC were determined taking into consideration a previous study [35], in which the justification was that biofilms were complex structures, and, therefore, higher concentrations of MIC would be necessary to obtain significant antifungal effect.

Indeed, for the biofilm of* C. albicans* higher concentrations of FBuTC than the MIC were necessary to reduce the number of viable cells of the biofilm when compared with the negative control group, whereas, for the biofilm of* C. glabrata*, the MIC was sufficient to obtain a significant reduction. Therefore, both species were susceptible to FBuTC; however, they responded with distinct susceptibility profiles. The antifungicidal activity observed in this study could be explained by the chemical composition of FBuTC [18, 29]. A previous study [23] characterized FBuTC by means of High Performance Liquid Chromatography, so that it was possible to establish that the main compounds present were hydrolyzable tannins (such as punicalin and punicalagin), gallic acid, and flavonoids. The mechanism of action of hydrolyzable tannins has been suggested to be directly related to the cell membrane, since these components are capable of precipitating proteins from the membrane and modifying the vital metabolic processes of the microorganisms [21]. Punicalin and punicalagin may possibly be the main compounds responsible for the antifungal activity observed [36, 37].

In addition to the hydrolyzable tannins, gallic acid and flavenoids are related to the inactivation of enzymes responsible for cellular adhesion, which is considered and important virulence factor of* Candida *spp. Moreover, they could impede the transport of proteins and cause rupture of the fungal cell [21, 23]. The possibility that there may be synergism of all the cited substances must not be discarded, and, in future studies, this may be an important basis for explanations about the mechanism of action of FBuTC.

Interestingly, in this study it was possible to observe that* C. glabrata *was more susceptible to FBuTC than* C. albicans. Candida* species are known to be capable of differing in terms of antifungal susceptibility and virulence factor profiles [15, 38].* C. glabrata*, for example, presents high levels of intrinsic and acquired levels of resistance to the azolic antifungal agents, while* C. albicans* is generally more susceptible to this type of treatment. On the other hand,* C. albicans* presents various characteristics of virulence that are absent in* C. glabrata*, such as the formation of hypha that play an important role in the formation of biofilms and tissue invasion [39].

Furthermore, differences in the hydrophobicity of the cell membrane have also been observed, and* C. albicans* is considered the most hydrophilic of the species [39, 40]. This difference in susceptibility may possibly be explained by the interactions of a hydrophobic nature that occurred in the cell membrane. Considering that FBuTC is an apolar fraction and that the cell membrane of* C. glabrata* is predominantly hydrophobic [15, 41], it could be speculated that greater interactions could be established on these surfaces, resulting in greater penetration of the tannins and flavenoids and consequently a greater antifungal effect. Thus, because of its hydrophilic nature, these interactions at the membrane level may possibly not be facilitated to the same extent in the biofilm of* C. albicans*, so that higher concentrations of FBuTC may be necessary for the antifungal effect to occur.

On the other hand, it has been demonstrated that the main disadvantages of using chemical and mechanical methods for cleaning dental prostheses are the changes in color and roughness that these products may induce in acrylic resin, which is directly related to the longevity and esthetics of the dental prosthesis [7, 24, 34, 42–46]. This is why the effect on color and roughness of acrylic resin was evaluated in this study, after immersion in (FBuTC).

Considering the color change values (ΔE), when compared with the group immersed in distilled water, statistical differences were observed for the immersions in both 1%SH and FBuTC, in all the time intervals of immersion. In spite of this variation, all the ΔE values obtained in the immersions in FBuTC were lower than 3.7 and were therefore, classified as clinically imperceptible [35]. The FBuTC possibly did not induce chemical reaction with the acrylic resin surface to the point of degrading its organic matrix and resulting in staining, which probably occurred in the 1%SH group as from the 21st day. Although the concentrations investigated were visibly of green color, it is speculated that their chromogenic molecules would be large enough to deposit them only on the resin surface [27]. As the immersion solution was changed daily, we believe that at each change these denser molecules were washed off, and, therefore, caused no visible color change. The effects of water on the property of acrylic resin color are relevant from the clinical point of view, because, frequently, the recommendation is to immerse dentures in water during the night (overnight immersion). However, in the period of time investigated, it was not possible to identify any change whatever.

Relative to the roughness values, FBuTC induced no significant changes in roughness when compared with the control group immersed in distilled water. This result is relevant, because roughness is a crucial factor for the adhesion of microorganisms on the acrylic surface. Therefore, it is of the utmost importance that chemical solutions do not change this property, because rough surfaces could favor the formation of biofilms [7, 24, 34, 42, 43]. Among the solutions for chemical control of biofilm, the 1%SH, used in an immersion protocol for 10 minutes, was considered the most efficient [34, 47]. In spite of these good results, when used in a prolonged regime it is capable of degrading the acrylic resin matrix causing a “bleaching effect” and causing the porosity of the surface [47], as confirmed in the present study.

Although 28 days of immersion of the resin in FBuTC do not represent the clinical reality and also appeared to be a short period of time in comparison with the useful life of a dental prosthesis, it is understood that the constant exposure to a solution that was changed every day could significantly age the acrylic matrix, creating a challenging situation for the tested material [35].

## 5. Conclusion

It could be concluded that the immersion of acrylic resin for dental prosthesis in FBuTC was effective in reducing the biofilms of* C. albicans* and* C. glabrata* without evidence of change in roughness and color of this substrate.

## Data Availability

The data used to support the findings of this study are included within the article.
